# Effects of Handgrip Strength on 10-Year Cardiovascular Risk among the Korean Middle-Aged Population: The Korea National Health and Nutrition Examination Survey 2014

**DOI:** 10.3390/healthcare8040458

**Published:** 2020-11-04

**Authors:** JaeLan Shim, Hye Jin Yoo

**Affiliations:** 1 Department of Nursing Gyeongju, College of Medicine, Dongguk University, Gyeongju 38066, Korea; jrshim@dongguk.ac.kr; 2 Department of Nursing, Asan Medical Center, Seoul 05505, Korea

**Keywords:** handgrip, cardiovascular disease, risk

## Abstract

Handgrip strength is a simple, inexpensive health status indicator and can be used to assess mortality rate and cardiovascular disease (CVD) risk. This study used data from the Sixth Korea National Health and Nutrition Examination Survey (2014) to determine the effective use of handgrip strength to predict CVD risk. We analyzed data from 2427 adults aged from 40 to 64 years without CVD at baseline. Relative handgrip strength was calculated as the sum of the maximal absolute handgrip strength of both hands divided by body mass index, and the 10-year risk of CVD was calculated using the Framingham risk score. We performed logistic regression analysis to assess the association between handgrip strength and 10-year CVD risk. Results showed that CVD risk increased with age (95% CI: 1.19–1.33, *p* < 0.001). Men were 38.05 times more likely to develop CVD than women (95% CI: 15.80–91.58, *p* < 0.001). Every increase by 1 in handgrip strength reduced the 10-year CVD risk by 1.76 times (95% CI: 1.58–3.71, *p* < 0.001), and when waist-to-height ratio was <0.50, the CVD risk decreased by 3.3 times (95% CI: 0.16–0.56, *p* < 0.001). Developing specific modifications and improving lifestyle habits that could lead to increased handgrip strength and reduced obesity, which could prevent CVD, is recommended.

## 1. Introduction

Cardiovascular disease (CVD) is a primary cause of death worldwide; it has been the leading cause of death in Korea for the past 10 years, with the highest mortality rate among single diseases besides cancer [1]. Addressing this burden on healthcare systems around the world requires an in-depth understanding of cardiovascular risk factors and the incidence and prevalence of various forms of CVD [2].

Handgrip strength (HGS) not only indicates the muscle strength of upper extremities but can also indicate that of the central core and lower extremities if measured in a standing position. It is also used to diagnose sarcopenia [3], and a better handgrip strength may be associated with cardiac functions and structures that help reduce the risk of cardiovascular incidents [4]. Other previous studies have also shown the association of HGS with frailty [5], falls [6], functional limitation [7], nutritional status [8], diabetes [9], osteoporosis [10], metabolic syndrome [11], and mortality rate [12,13]. HGS varies by country; HGS among Koreans is strongest among those in their late 30s and begins to decrease after they enter their 40s [14].

In women, climacterium and pre- and post-menopause usually occur during middle age, along with rising glucose levels and increases in blood pressure, reinforcing the importance of taking preventive measures for CVD during this period [15]. However, according to nationwide statistics on causes of death, CVD’s mortality rate is increasing particularly among people over the age of 40 [1]. Moreover, the health conditions that affect the development of CVD, such as diabetes, hypertension, and hyperlipidemia, are poorly managed [16]. This suggests the need to develop a proactive strategy to reduce the prevalence of CVD among the middle-aged population. The risk factors for CVD are already known, namely increased body mass index (BMI), dyslipidemia, and lack of physical activity [17]. In addition, among the obesity indicators, the waist-to-height ratio (WHtR), which is highly correlated with cardiovascular risk factors and reflects the distribution of visceral fat among Asians, predicts cardiometabolic disease, which measures the degree of central obesity as a useful CVD risk screening tool [18]. As such, research on CVD risk factors has been steadily progressing, but few studies have dealt with these various factors altogether.

The Framingham risk score (FRS) is a simple and standardized tool to evaluate the 10-year risk of coronary artery disease and to predict an individual’s long-term chances of developing CVD [19]. It is used to assess CVD risk in individuals who have not yet developed CVD by considering various related factors and serves as a guideline for risk factor management [20]. Although HGS measurement is not regularly performed during doctor visits, if HGS can predict the risk of CVD or death caused by CVD, it can be a useful tool that can be easily utilized in clinical settings. Few studies have found causative relationships between HGS and CVD risk factors, but this is because they measured the HGS of the dominant hand [21,22].

This study used a nationally representative sample from the Korea National Health and Nutrition Examination Survey (KNHANES) to examine the effectiveness of using HGS, excluding the CVD risk factors that are identified so far, to predict CVD risk among middle-aged subjects who have a high prevalence of CVD, as measured by the FRS. These data can be utilized to design a future program to prevent the development of CVD. Nevertheless, a longitudinal study that can reveal the causal relationship between cardiovascular disease and HGS in the future is needed.

## 2. Materials and Methods 

### 2.1. Study Design

This secondary data analysis adopted a cross-sectional correlational study design to confirm CVD risk and the impact of HGS for Korean middle-aged participants using data from the KNHANES conducted in 2014. 

### 2.2. Setting and Sample

The KNHANES is a government-approved statistical survey conducted by the Korea Disease Control and Prevention Agency (KDCA) (Ethical approved project identification code. 2013-12 EXP-03-5C). It was established to monitor the health and nutritional status of Koreans every year since 1998 [23]. The KNHANES collects data on participants’ demographics and social, health, and nutritional status using three component surveys: health interviews, health examinations, and nutrition surveys.

**Figure 1:**
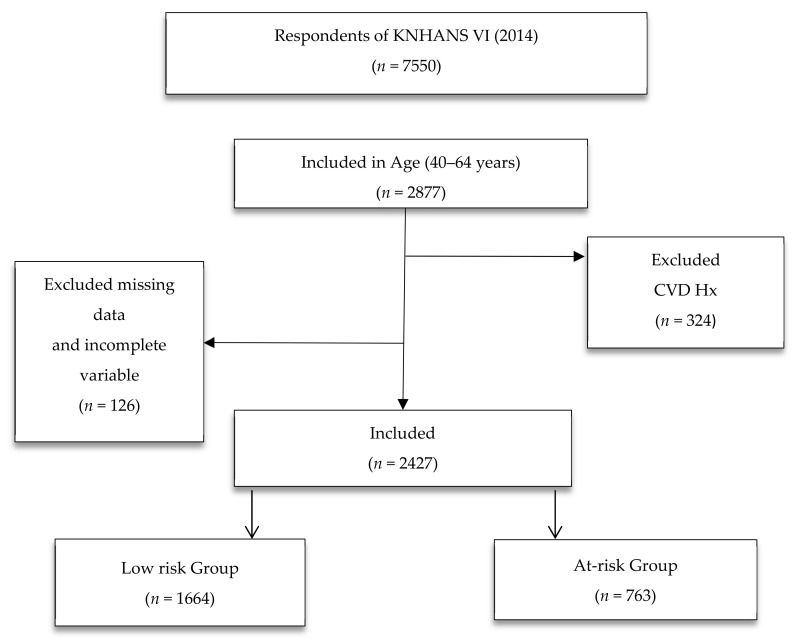
Flow chart of the study population. Abbreviations: CVD, cardiovascular disease; KNHANES VI, Korea National Health and Nutrition Examination Survey VI.

The current study received approval for data use by accessing the KDCA website [24]. We downloaded the Sixth KNHANES questionnaires and results in SPSS format. We excluded participants aged between 40 and 64 years with a history of CVD (*n* = 324), and those with insufficient information (*n* = 126). As a result, 2427 of the total 7550 participants were included in this study (Figure 1).

### 2.3. Measurements

#### 2.3.1. The FRS 

The FRS calculation followed the method suggested by Wilson et al. [25] from the Framingham Heart Study and considered sex, age, low-density lipoprotein (LDL) cholesterol level, high-density lipoprotein (HDL) cholesterol level, total cholesterol level, systolic blood pressure, diastolic blood pressure, smoking status, and diabetes existence. According to this model, each of the following factors was categorized into subgroups that were divided between men and women and assigned a respective risk score: age (nine subgroups; those aged from 30 to 34 years start with zero points, while those aged from 35 to 39 years start with two points. An additional two points are given each time age increases by five years.), HDL cholesterol level (those with 1.6 mg/dL or more start with minus two points, and an additional one point is given for every increase of 0.3 mg/dL), total cholesterol level (those with 4.1 mg/dL start with zero points, and an additional one point is given for every increase of 1 mg/dL), systolic blood pressure (five subgroups; for those with a systolic blood pressure of 130 to 139 mmHg, three points are given for taking antihypertensive drugs, while one point is given for not taking antihypertensive drugs. An additional one point is given for every 10 mmHg increase in systolic blood pressure from the baseline), diabetes (those without diabetes start with zero points, while those with diabetes start with three points), and smoking (those who do not smoke start with zero points, while men and women who smoke start with four and three points, respectively). We took the factors into account according to the following steps: age in step one, either LDL cholesterol level or total cholesterol level in step two, HDL cholesterol in step three, systolic blood pressure in step four, diabetes in step five, and smoking in step six. We assigned different risk scores to total cholesterol level, which has a total of nine factors, throughout the six steps. We then calculated the 10-year risk of CVD using both the sum scores of the calculated levels of LDL cholesterol only, and of the calculated levels of LDL cholesterol and total cholesterol together [25]. A risk score of <10% is considered very low risk, 10% to 20% an intermediate risk, and >20% a high risk for 10-year cardiovascular events [25]. In this study, an FRS of <10% was defined as a low-risk group, while ≥10% was defined as an at-risk group.

#### 2.3.2. HGS

HGS is the force of grip applied by the four fingers and thumb when squeezing any object. HGS was measured using a digital hand dynamometer (T.K.K. 5401; Takei Scientific Instruments Co., Tokyo, Japan) before performing other examinations such as pulmonary function testing as well as blood sample collection, which could have affected the result. The researchers who conducted the Sixth KNHANES provided participants with adequate information regarding test instruction and precautions. Participants were instructed to perform the test in a relaxed and comfortable position; those who had hand or wrist surgery within the last three months and experienced pain, soreness, or stiffness in their hands within the last seven days prior to the examination were excluded. Before the measurement process, the participants performed light exercise by squeezing all 10 fingers after removing all jewelry on their fingers and wrists, which they repeated thrice. The measurements were collected while the participants were standing, looking straight ahead, and keeping their back straight. Knuckles were perpendicular to the dynamometer’s handgrip, and the maximum squeezing time was less than three seconds [26].

Absolute HGS was calculated by summing the highest HGS of both hands. Relative HGS was used for analysis and was calculated by dividing the absolute HGS by the body mass index, (BMI) [27].

#### 2.3.3. Level of Physical Activity Assessment

The International Physical Activity Questionnaire-Short Form (IPAQ-SF) is a questionnaire tool developed by the International Consensus Group for the Development of an International Physical Activity Questionnaire in the World Health Organization [28]. It has been translated into five languages, and its validity and reliability have been verified through multinational studies [29].

The level of physical activity was assessed by collecting data from the Korean Survey of IPAQ-SF. Participants responded with the duration and number of days they performed high-intensity exercise for longer than 10 min as well as moderate-intensity exercise, such as walking, within the last seven days prior to participating in the survey. Respondents to the questionnaire were asked regarding their activity time using activity intensity.

Their answers included a predetermined metabolic equivalent task (MET, minutes) value for each activity intensity (sleeping = 1 MET, activity on sitting = 1.8 METs, light intensity activity = 2 METs, walking = 3.3 METs, moderate intensity activity = 4.0 METs, and vigorous intensity activity = 8.0 METs) [30,31]. The following conversion process was used to calculate the MET score from the assessment.

Amount of walking = 3.3 MET × duration (min) × number of days

Amount of moderate-intensity exercise = 4.0 MET × duration (min) × number of days

Amount of high-intensity exercise = 8.0 MET × duration (min) × number of days

Total amount of physical activity (MET min/w) = amount of walking + amount of moderate-intensity exercise + amount of high-intensity exercise.

The calculated total amount of physical activity was categorized into three groups based on the IPAQ scoring system [28,30,31]; the groups were then compared. The low-intensity exercise group included those who performed minimal or no physical activity compared to the moderate- and high-intensity exercise groups. Participants in the moderate-intensity exercise group included anyone who exercised at high intensity for at least 20 min per day for more than three days per week, at moderate intensity for at least 30 min per day for more than five days per week, walked for at least 30 min per day for more than five days per week, or exceeded 600 MET min/w with any combination of moderate- or high-intensity exercises. The high-intensity exercise group included anyone who exceeded 1500 MET min/w by performing high-intensity exercise for more than three days per week, walked seven days per week, or exceeded 3000 MET min/w with any combination of moderate- or high-intensity exercises [30,31,32].

#### 2.3.4. Central Obesity

A WHtR cutoff point of 0.50 [33] was obtained from measuring waist circumference and height. Fasting blood sugar, glycated hemoglobin, total cholesterol, triglyceride, high-density cholesterol, and low-density cholesterol levels as well as blood pressure were measured by nurses for analysis with respect to the blood test items of the KNHANES.

### 2.4. Analysis 

The collected data were analyzed using SPSS for Windows 23.0 (IBM Corp., Armonk, NY, USA). According to the guidelines for the use of raw data from the KNHANES, composite sample analysis was performed by taking sample weights into account. General characteristics of the population were organized as descriptive statistics, and the analytic hierarchy process was applied to all sample and weight variables. Each participant was statistically assigned a weight so that they could be selected with the same probability, and so that the result would be representative of all Koreans. Sampling weights were applied to illustrate KNHANES’ complex sampling method, which included stratification. Stratification by gender and age was conducted first, followed by stratification by sex and age. To avoid bias caused by changes in age and sex, distribution, age, and sex were adjusted and applied to the 2014 Korean population.

Logistic regression analysis was used to determine the correlation between HGS and the degree of CVD risk, and the significance level was set at *p* < 0.05. Multiple imputations for missing values were performed for continuous variables using mean values.

## 3. Results

### 3.1. General Characteristics of Study Participants

This study included a total of 2427 participants; 1664 participants were in the low-risk group and 763 were in the at-risk group. The average age of participants in the low-risk group was 49.31 (0.21) years, and 54.92 (0.27) years for the at-risk group; there was a statistically significant difference between the two groups (*p* < 0.001). Statistically significant differences were also found between the two groups based on marriage status, residential setting, education level, household income, occupation, drinking status, smoking status, and perceived health status (Table 1).

### 3.2. Differences in HGS and CVD Risk Factors between the Two Groups

According to the FRS, the HGS in the low-risk group was 36.79 (0.36) kg and 29.94 (0.28) kg in the at-risk group. The at-risk group showed weaker HGS, and there was a statistically significant difference between the two groups (Table 2).

**Figure 2:**
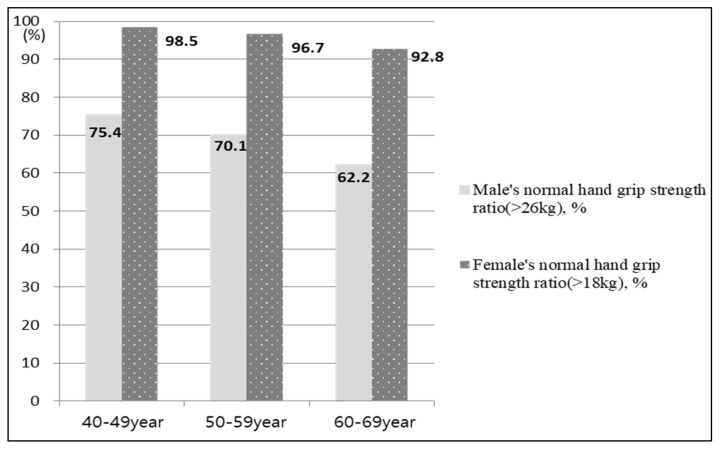
Differences in male and female normal grip strength ratio according to age.

As shown in Figure 2, according to the Asian Working Group of Sarcopenia [34], the average HGS is 26 kg in men and 18 kg in women. We compared the average HGS among the participants in the current study to this standard. As a result of checking the change in HGS by age group, the HGS of women was decreased by about 2%, and by up to 4% as age increased. Moreover, the HGS of men was decreased by about 8%, and by up to 5% as age increased, which was greater than that of women. For obesity-related BMI, the at-risk group had a significantly greater number of subjects (BMI > 25 kg/m^2^; *p* < 0.001). In terms of the mean WHtR as well as the fasting glucose and HbA1c tests, the at-risk group had a high value and levels, respectively, showing a significant difference with the low-risk group (*p* < 0.001). For the lipid test, the at-risk group scored high except for LDL, showing a difference between the two groups (Table 2).

### 3.3. Factors Influencing the 10-Year Risk of CVD

Logistic regression analysis was used to identify factors influencing CVD risk (Table 3). Goodness of fit was significant (χ^2^ = 41.47, *p* < 0.001), with Negelkerke R^2^ = 13.8%. The following independent variables were added to the logistic regression analysis: age, sex, HGS, physical activity, WHtR, and sedentary time; the results of logistic regression analysis showed that the risk of developing CVD increased by 1.26 times as age increased (95% CI: 1.19–1.33, *p* < 0.001).

Men were more likely to develop CVD than women by 38.05 times (95% CI: 15.80–91.58, *p* < 0.001). The 10-year risk of CVD decreased with stronger HGS. Every increase by one in HGS decreased the 10-year risk of CVD by 1.76 times (95% CI: 1.58–3.71, *p*< 0.001), and a WHtR <0.50 reduced the risk of CVD by 3.3 times (95% CI: 0.16–0.56, *p* < 0.001).

## 4. Discussion

Utilizing data obtained from the Sixth KNHANES in 2014, this study used the FRS to estimate the risk of CVD among the Korean middle-aged population. CVD risk factors were identified as age, sex, HGS, and WHtR. We found that CVD risk increased as age increased. This result correlates with a study in which CVD risk factors and age showed a positive linear relationship [35]. Between 4% and 10% of cardiac arrests occur in men younger than 45, which indicates that younger people are at risk of developing CVD despite their young age. Thus, our result reinforces the importance of early prevention, as atherosclerosis can begin to develop during adolescence [36].

Our results also showed that the risk of developing CVD varied by sex: men were 38 times more likely to develop CVD than women. Examination of the incidence of ischemic heart disease (IHD) showed that among adult patients over the age of 30, men were 2.38 times more likely to develop IHD than women [37]. According to a study on elderly patients over the age of 75 with acute myocardial infarction [38], there was no significant difference in hospital-acquired complication or mortality rate by sex. However, sex was identified as an independent factor that predicted one-year major cardiac adverse events, and men had worse prognoses than women (HR 1.37, 95% CI: 1.14–1.65). According to Sohn et al. [39], the categorization of CVD risk factors based on FRS is associated with blood interleukin-6 concentration; Korean men’s lifestyles, including a high-cholesterol diet, alcohol consumption, and smoking, are major causes of metabolic syndrome and are useful in predicting CVD risk [39]. In particular, the participants of this study were middle-aged, which is the period when they play an active, major role in economic production, resulting in work-related stress and challenges that may affect lifestyle behaviors, including poor diet and smoking. Therefore, a specific strategy to modify CVD-related lifestyle behaviors is necessary.

This study identified HGS as a powerful predictive factor of CVD risk, excluding age. Data analysis of 14,000 individuals from the multicultural prospective urban-rural epidemiology study supported the current study’s results that HGS is an excellent indicator, on par with systolic blood pressure, for predicting CVD-related death [40]. According to a study conducted in Switzerland with 3468 adults aged 50 to 75 years, HGS had a moderate association with CVD risk indicators [41]. Grip strength has been proposed as a biomarker that predicts the mortality rate of specific diseases, future functionality, bone density, fractures, cognition and depression, and hospitalization-related problems [41]. Weak grip strength is associated with subsequent disability and mortality [42], and weakened HGS was a significant risk factor of adult mortality rate in men and women, with stronger significance in men [43]. However, it is difficult to apply an HGS standard value set by a specific country to another country: standard values based on Western participants are not suitable for use in Asia. Therefore, it is recommended to use a suitable HGS for each country [42]. Bae, Park, Sohn, and Kim [43] suggested an adequate Korean HGS for optimal health as a minimum of 16.8 kg for women and 32.8 kg for men, and reported a strong causative relationship between weakened HGS and high mortality rate. Predicting the risk of CVD through easy assessment and monitoring of HGS has noteworthy potential in public healthcare and could contribute to a decrease in CVD-caused mortality rate. In addition, taking the standard HGS for Koreans into account when developing exercise interventions for CVD prevention would be a useful strategy to help Koreans maintain healthy hearts.

In this study, men showed a sharper decrease in HGS as age increased compared to women. This is similar to the result of a study investigating the change in grip strength of men and women over 30 years old using the Mini-Finland Health Examination Survey, which was 3.5 Newtons per year for men and 2.0 Newtons for women [44]. In addition, physical well-being, especially muscle strength (estimated by HGS), is an important indicator of wellness in old age as it leads to better health-related quality of life [45]. Therefore, since lower HGS might be a factor that leads to deterioration in physical quality of life [45], men should seek ways to strengthen handgrip starting at a young age.

This study shows that individuals with a WHtR above 0.5 had a higher CVD risk than those with a WHtR below 0.5. WHtR has been found to be indicative of heart problems in Western, Asian, and Central American countries and is a stronger CVD predictive variable than BMI. In this study, the average systolic and diastolic blood pressure, as well as glucose levels, were higher in the at-risk group than the low-risk group. In previous studies, there was a correlation between fasting glucose levels and FRS [46], and it was confirmed that CVD risk was high in the hypertension category even among adolescents and the middle-aged [47], as supported by the present study.

According to a large-scale study with middle-aged men [48], BMI, total cholesterol level, blood pressure, and fasting glucose increased together with average WHtR; however, there was a negative correlation between WHtR and cardiovascular health scores. Further, WHtR, as a predictor of CVD risk, may be more precise than BMI, weight, or waist circumference [49,50,51]. A previous study [52] implemented a diet modification based on Korean cuisine with participants for 12 weeks; the results showed a significant decrease in animal fat and cholesterol intake, thus reducing the risk of CVD. Therefore, education and health campaigns recommending a Korean cuisine-based diet, which has lower fat content and is more nutritionally balanced than a dairy- and protein-heavy Western diet, should be implemented. Additionally, weight management through regular exercise can be recommended to reduce the risk of CVD.

It should be noted that the current study’s results showed that physical activity and sedentary time were not valid or significant predictive factors of 10-year CVD risk; this result contrasts with previous studies that have identified their association with CVD risk [53,54,55]. The KNHANES measures physical activity with the IPAQ-SF translated into Korean. However, the IPAQ does not consider walking a moderate-intensity exercise, unlike other surveys such as the National Health Interview Survey or Behavioral Risk Factor Surveillance System. This could explain why physical activity was not associated with the risk of developing CVD in this study. Furthermore, research on sedentary time and CVD risk has shown an increased risk of CVD and mortality rate with increased sitting time, including time spent watching TV, total time spent in a sitting position, and time spent sitting in cars [56]. However, sedentary time was assessed using a self-report survey with the following question: “how long do you spend sitting or lying down in a day” with participants responding in hours and minutes. Therefore, future studies should use a more detailed and objective tool for assessment since a self-report survey does not objectively assess this criterion. Moreover, research by more qualified experts should be conducted to secure its reliability and effectiveness.

Physical activity is closely related to CVD incidence. The level of physical activity begins to decrease among individuals aged from 50 to 65 years, which leads to the development of chronic conditions and an increased mortality rate. Therefore, increasing the level of physical activity of the middle-aged population is extremely important [16]. Although there is an ongoing debate about the appropriate amount and intensity of exercise [54], 30 min of daily moderate-intensity exercise may help reduce CVD risk in the following ways: it may increase insulin sensitivity and help control blood sugar level, and it positively influences the anti-inflammatory response, obesity, blood pressure, cholesterol level, and endothelial function [53,55]. It is especially recommended to conduct over 75 min of high-intensity exercise or over 150 min of moderate-intensity exercise per week to prevent cardiovascular and cerebrovascular diseases [57]. This suggests that exercise is necessary for CVD prevention. Further, despite an increase in physical activity level, if sedentary time also increases, the mortality rate would increase [58]. Longer periods of sedentary time lead to an increase in mortality rate, obesity, diabetes, metabolic syndrome, and CVD [57]. Therefore, it is important to develop a strategy to increase physical activity while reducing sedentary time. It is recommended to achieve the recommended level of daily physical activity every day, but many individuals are unable to do this because of their busy schedules. Therefore, it is crucial to perform safe, low-intensity exercises such as walking to prevent CVD [54]. It is also necessary to educate the public on the importance of practicing this in their daily lives.

This study has several limitations. First, a cross-sectional study cannot identify influencing factors and determine the sequence of events. Second, although the survey questionnaires were created by skilled instructors and researchers, this study is based on a self-report survey questionnaire with no objective measurement index. There is a possibility that participants’ responses were subjective and biased depending on their social background. Further, since this is a retrospective assessment, measurement bias cannot be ruled out.

Despite these limitations, this is a national study that analyzed data from all citizens using a standardized method for assessment. A national representative sample as well as FRS, which can predict 10-year CVD risk in middle-aged individuals with no CVD, were used to identify the correlation between HGS and CVD. As HGS has been identified as a simple, easy tool to predict the risk of CVD development, we recommend conducting a longitudinal study that can trace the incidence of CVD after improving HGS.

## 5. Conclusions

This study included adults aged from 40 to 64 years with no CVD, and identified the risk factors of FRS-calculated CVD as age, sex, HGS, and WHtR. Among these risk factors, HGS was identified as the most influential factor of CVD risk, except for sex. The middle-aged population plays an active role in economic production, and the identification of the 10-year risk factors of CVD would help in the early prevention of the disease, thus reducing mortality rate and healthcare expenses. Therefore, this finding is significant to the public healthcare system. It is recommended to develop a realistic management program that can strengthen HGS enough to minimize risk of CVD and promote diet modification and regular exercise.

## Figures and Tables

**Table 1:** Unweighted frequencies of demographic and health-related characteristics of the middle-aged, KNHANES 2014.

Variable	Low Risk (<FRS 9%) (*n* = 1664)		At-Risk * (≥FRS 10%) (*n* = 763)		*p*
	*n* or Estimates % (SE)				
Age (years), mean (SE)	49.31 (0.21)		54.92 (0.27)		<0.001
Age, *n* (%)					<0.001
40–54	1230	76.9 (1.1)	385	50.5 (0.8)	
55–64	434	23.1 (2.0)	378	49.5 (0.6)	
Sex					<0.001
Male	379	31.0 (1.2)	576	83.2 (1.3)	
Female	1285	69.0 (1.2)	187	16.8 (1.3)	
Marital status					<0.001
Married	1435	87.8 (0.9)	664	87.8 (1.6)	
Divorced/Unmarried	167	8.2 (0.8)	75	8.8 (1.2)	
Others	62	4.0 (0.4)	24	3.4 (0.8)	
Residential area					<0.001
Urban	1397	84.1 (2.8)	596	79.8 (3.1)	
Rural	267	15.9 (2.8)	167	20.2 (3.1)	
Education					<0.001
<Elementary school	211	11.7 (1.0)	188	22.6 (2.1)	
Middle school	200	12.5 (1.0)	121	17.1 (1.7)	
High school	598	44.1 (1.8)	213	31.6 (2.2)	
≥College	440	31.7 (2.0)	171	28.8 (2.5)	
No response	215		70		
Household income					<0.001
1st quartile	152	8.6 (0.9)	123	14.0 (1.5)	
2nd quartile	417	23.7 (1.6)	199	25.2 (1.8)	
3rd quartile	538	33.3 (1.7)	225	30.2 (2.0)	
4th quartile	548	34.4 (2.2)	214	30.6 (2.5)	
Occupation					<0.001
Managerial and professional	329	20.8 (1.2)	136	21.0 (2.1)	
Service and sales	271	16.7 (1.2)	78	10.2 (1.3)	
Routine and manual	326	19.5 (1.3)	305	39.4 (2.4)	
Unemployed	738	42.9 (1.5)	244	29.4 (2.2)	
Frequency of drinking					<0.001
None	280	15.2 (1.1)	100	11.9 (1.2)	
1/week	810	48.8 (1.3)	286	36.2 (2.1)	
2–3/week	191	12.0 (0.9)	157	24.2 (2.0)	
Daily	383	24.0 (1.2)	220	27.7 (1.9)	
Smoking					<0.001
Current smoker	1559	7.5 (0.7)	345	51.9 (2.1)	
Ex-smoker/Never	105	92.5 (0.7)	418	48.1 (2.1)	
Perceived health status					<0.001
Poor	447	28.0 (1.4)	208	27.1 (2.0)	
Moderate	788	45.9 (1.5)	377	49.2 (2.3)	
Good	429	26.0 (1.2)	178	23.7 (1.7)	

SE, standard error; FRS, Framingham risk score; KNHANES, Korea National Health and Nutrition Examination Survey; * at-risk includes the intermediate or high cardiovascular risk group with FRS of 10% or greater.

**Table 2:** Clinical characteristics and biomarkers of the subjects by FRS.

Variable	Low Risk (<FRS 10%)	At-Risk (≥FRS 10%)	*p*
	Estimates % (SE)	Estimates % (SE)	
Handgrip strength (kg), mean (SD)	36.79 (0.36)	29.94 (0.28)	<0.001
Sedentary time (hr/d), mean (SD)	6.39 (0.14)	6.83 (0.20)	0.054
Physical activity (min/w), mean (SD)	671.96 (0.32)	632.40 (0.50)	<0.001
Light PA	59.8 (0.3)	63.5 (0.3)	
Moderate PA	36.9 (0.2)	33.8 (0.4)	
Vigorous PA	3.3 (0.3)	2.7 (0.3)	
Body mass index (kg/m^2^), mean (SD)	23.55 (0.92)	24.86 (0.12)	<0.001
<25	71.8 (0.4)	52.7 (0.3)	
≥25	28.2 (0.3)	47.3 (0.3)	
Waist to height ratio, mean (SD)	0.49 (0.00)	0.52 (0.00)	<0.001
<0.50	50.8 (0.3)	32.4 (0.3)	
≥0.50	49.2 (0.2)	67.6 (0.2)	
Blood pressure (mmHg)			
Systolic	112.63 (0.36)	127.88 (0.83)	<0.001
Diastolic	75.04 (9.21)	81.67 (10.16)	<0.001
Fasting glucose (mg/dL), mean (SD)	98.24 (0.58)	110.52 (1.17)	<0.001
Cholesterol (mg/dL), mean (SD)	192.24(0.01)	199.51 (0.02)	<0.001
Total cholesterol, mean (SD)	192.24 (0.96)	198.86 (1.77)	<0.001
Triglyceride	122.42 (2.84)	200.04 (5.34)	<0.001
HDL cholesterol	53.51 (0.28)	45.90 (0.42)	<0.001
LDL cholesterol	121.18 (3.71)	118.87 (2.61)	0.598
HbA1c (%)	5.68 (0.02)	6.12 (0.04)	<0.001

SE, standard error; FRS, Framingham risk score; KNHANES, Korea National Health and Nutrition Examination Survey; SD, standard deviation; PA, physical activity; HDL, high-density lipoprotein; LDL, low-density lipoprotein; HbA1c, hemoglobin A1c.

**Table 3:** Factors affecting cardiovascular risk according to FRS.

Variables	Categories	Low Risk vs At-Risk for CVD	
		OR (95% CI)	*p*
Age	≥50	1.26 (1.19–1.33)	<0.001
	40–49	1	
Sex	Male	38.05 (15.80–91.58)	<0.001
	Female	1	
Handgrip strength		−1.76 (1.18–3.71)	<0.001
Physical activity (min/w)	Light	1.48 (0.25–8.58)	0.669
	Moderate to Vigorous	1	
Waist to height ratio	<0.50	0.30 (0.16–0.56)	<0.001
	≥0.50	1	
Sedentary time (hr/d)	M ± SD	0.96 (0.88–1.04)	0.265
χ^2^ = 41.47, *p* < 0.001, Negelkerke R^2^ = 13.8			

CVD, cardiovascular disease; CI, confidence interval; OR, odds ratio; M, mean; SD, standard deviation.
